# False Elevation of Volume Determined by Bladder Scanner Secondary to Bowel Obstruction

**DOI:** 10.5811/cpcem.2019.12.45103

**Published:** 2020-02-24

**Authors:** Sean Schowalter, Zaid Altawil, Elissa M. Schechter-Perkins, Joseph R. Pare

**Affiliations:** * Beth Israel Deaconess Medical Center, Department of Internal Medicine, Boston, Massachusetts; † Boston Medical Center, Department of Emergency Medicine, Boston, Massachusetts

## Abstract

Bladder scanners allow for quick determination of bladder volumes (BV) with minimal training. BV measured by a machine is generally accurate; however, circumstances exist in which falsely elevated BVs are reported. This case details a patient with a significant small bowel obstruction (SBO) due to superior mesenteric artery syndrome causing a falsely elevated BV. We believe this is the first case report of a SBO causing an elevated BV by bladder scanner. Emergency physicians should be aware of the pitfalls of using bladder scanners, and use their point-of-care ultrasound skills when possible to expand their differential.

## INTRODUCTION

Bladder scanners have seen widespread use in emergency departments (ED) due to their ease of use and relatively low cost of application.^1^ They can be used by registered nurses (RN), and are helpful in determining the presence of acute urinary retention, saving patients the discomfort of undergoing unnecessary bladder catheterization.^1^ However, their use is not without diagnostic pitfalls. Previous case reports have documented inaccurate bladder volume (BV) measurements attributed to pelvic structures such as ovarian cysts and uterine myomas.^2^,^3^,^4^,^5^ In the following case, we present a previously unreported cause of falsely elevated BV due to abdominal pathology.

## CASE REPORT

A 56-year-old female with a history of latent tuberculosis, stage IIIA non-small cell lung cancer status post right lower and middle lobectomy and lymph node dissection four months prior, was transferred from an outside hospital with concern for sepsis. She spoke only Mandarin; limited history was provided by her daughter at the bedside. Her daughter reported that the patient came to the hospital after two episodes of syncope. Her review of systems was positive for one week of worsening nausea, abdominal discomfort, poor oral intake, several episodes of bilious vomiting, and difficulty urinating. She had not had a bowel movement in the prior three days but was passing flatus. Abnormal vital signs at the outside hospital included a blood pressure of 80/53 millimeters of mercury (mmHg) and a heart rate of 124 beats per minute.

Relevant abnormal laboratory results from outside hospital records included a leukocyte count of 16.7×10^9 cells per liter (L) (reference: 4.0–11.0 ×10^9 cells/L), a lactate of 6.1 millimoles (mmol)/L (reference: 0.9–1.7 mmol/L), and a sodium of 124 milliequivalents (mEq)/L (reference: 135–145 mEq L). Her electrocardiogram was significant for sinus tachycardia. Outside hospital (OSH) chest radiography revealed unchanged chronic right hydropneumothorax without infiltrate, and an OSH abdominal radiograph report showed no evidence of an ileus or an obstruction. Prior to arrival at our institution, the patient received two liters of normal saline along with intravenous vancomycin, ceftriaxone and metronidazole.

Upon examination in our ED she was noted to be afebrile, and her blood pressure had improved to 97/54 mmHg. Her tachycardia had resolved. The patient appeared chronically ill and lethargic with a tense, diffusely tender abdomen. Repeat laboratory investigations demonstrated resolution of her elevated lactate and stable leukocytosis. A tentative diagnosis of urosepsis was made.

**Image 1:**
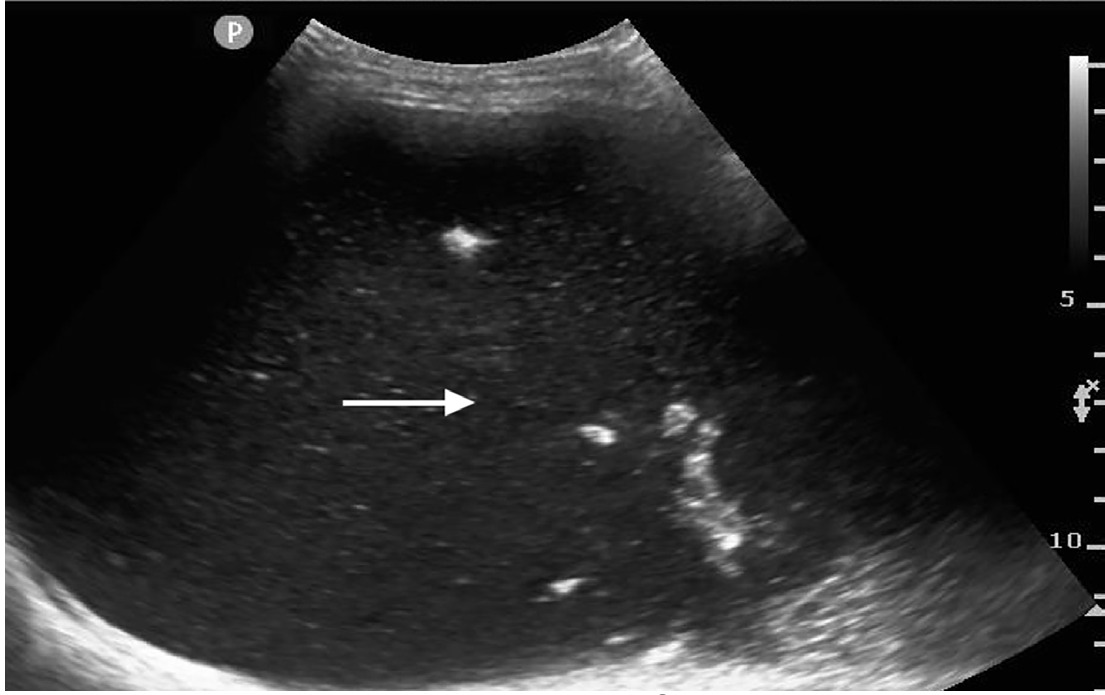
Transverse ultrasound view of abdomen demonstrating a large amount of fluid and fecal matter within a massively distended loop of bowel. Arrow is within a distended loop of bowel.

Given the need for a urine sample and concern for urinary retention per history, an RN performed a bladder scan in preparation for catheterization, which estimated a volume of 900 milliliters (mL). A Foley catheter was placed with removal of 600 mL of urine. On repeat bladder scan, a value of 900mL was again obtained. Discordance between bladder scan and catheterization prompted the physician to perform a point-of-care ultrasound (POCUS), which showed severely dilated bowel loops filled with fluid, concerning for a small bowel obstruction (SBO) (Image 1).

**Image 2:**
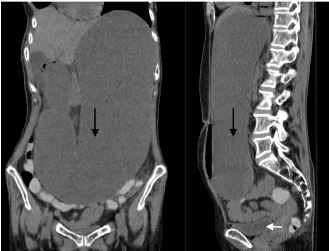
(a) Coronal computed tomography(CT) image demonstrating a markedly distended loop of bowel; (b) Sagittal CT image demonstrating a markedly distended loop of bowel, as well as partial view of the bladder. Black arrows represent the loop of bowel. White arrow points to the bladder.

A computed tomography (CT) was ordered to confirm the diagnosis and showed marked fluid distention of the distal esophagus, stomach, and proximal duodenum to the level of the superior mesenteric artery (SMA) consistent with a SBO (Image 2). Given the distribution, findings were attributed to SMA syndrome.

A nasogastric tube was placed, which returned three liters of fecalized fluid. The patient was admitted under the general surgery service. She underwent initial lysis of her ligament of Treitz, and ultimately required duodenal jejunostomy to treat her obstruction.

## DISCUSSION

Dedicated bladder scanners are a useful, non-invasive, and accurate tool for the evaluation of patients with suspected urinary retention.^1^ By incorporating automated algorithms that calculate BVs, these machines have become easier to use and are essentially user-independent.^6^ This allows novice operators, including RNs, to be easily and quickly trained in their use. However, this convenience has also come at the cost of specificity, with false positive rates cited as high as 9%.^2^,^7^

Possible reasons for falsely elevated BVs include ovarian and renal cysts,^2^,^4^ uterine myomas,^3^ and ascites.^5^ These elevations are likely due to an inability of bladder scanners to differentiate between fluid in the bladder and other hypoechoic areas in the pelvis. This has important implications, as falsely elevated BVs can lead to unnecessary catheterizations and a delay in diagnosis, as was evident in this case. Fortunately, the physician was able to use POCUS to visualize the bladder and adjacent structures to suggest the alternate diagnosis of SBO and expedite management. POCUS has been previously shown to have high sensitivity (94–100%) and specificity (81–100%) for the detection of SBO.^8^ While the current gold standard for diagnosing SBO is CT, diagnosis requires an elevated index of suspicion, and delays in obtaining CT imaging may occur.^9^ As shown in the case, POCUS was used to resolve diagnostic inaccuracies by bladder scanner and abdominal radiography.

**CPC-EM Capsule**

What do we already know about this clinical entity?

There are many causes of falsely elevated bladder volumes on bladder scanners including ovarian cysts, renal cysts, ascites, or uterine myomas.

What makes this presentation of disease reportable?

This was the first report of falsely elevated bladder volume due to small bowel obstruction.

What is the major learning point?

Falsely elevated volumes as measured by bladder scanners occurs in up to 9% of bladder scans.

How might this improve emergency medicine practice?

Although bladder scanners are an extremely useful tool, physicians should recognize that there are several causes of falsely elevated bladder volume.

## CONCLUSION

This case represents the first report of a falsely elevated bladder volume by bladder scanner attributed to a bowel obstruction. This emphasizes the importance of further workup in cases of discordance between volumes obtained by bladder scanner and catheterization. SBO is a potentially life-threatening condition and a delay in diagnosis can lead to increased morbidity and mortality.^10^ Therefore, physicians should be aware of the pitfalls of routine automated bladder scanners, and use their POCUS skills when possible to expand their differential.
